# Upfront fixed-duration treatment strategies for chronic lymphocytic leukemia in Arab populations: a position statement from the Gulf region

**DOI:** 10.3389/fmed.2025.1509074

**Published:** 2025-02-26

**Authors:** Mohamed A. Yassin, Khalil Al Farsi, Anas Hamad, Rola Ghasoub, Ahmad Alhuraiji, Kayane Mheidly, Hasan Aal Yaseen, Hani Osman, Martin Trepel

**Affiliations:** ^1^Department of Medical Oncology/Hematology, National Centre for Cancer Care and Research, Hamad Medical Corporation, Doha, Qatar; ^2^Department of Hematology, Sultan Qaboos University Hospital, Muscat, Oman; ^3^Department of Pharmacy, National Center for Cancer Care and Research, Hamad Medical Corporation, Doha, Qatar; ^4^College of Pharmacy, QU Health, Qatar University, Doha, Qatar; ^5^Department of Hematology, Kuwait Cancer Control Center, Kuwait City, Kuwait; ^6^Translational Research Department, Dasman Diabetes Institute, Kuwait City, Kuwait; ^7^Department of Medicine, Division of Hematology, Sheikh Shakhbout Medical City, Abu Dhabi, United Arab Emirates; ^8^Department of Hematology, Dubai Hospital, Dubai, United Arab Emirates; ^9^Department of Hematology, Tawam Hospital, Al Ain, Abu Dhabi, United Arab Emirates; ^10^Department of Hematology and Oncology, Faculty of Medicine, Augsburg University Hospital, University of Augsburg, Augsburg, Germany

**Keywords:** chronic lymphocytic leukemia, fixed-duration treatment, ibrutinib, venetoclax, obinutuzumab, Arab, Middle East

## Abstract

The treatment landscape for chronic lymphocytic leukemia (CLL) has expanded dramatically over the last decade, with a wide range of effective treatments now available. Clinical management of CLL varies widely depending on patient profile, meaning the optimal treatment in Arab patients, who tend to be young and often present with comorbidities, including diabetes and obesity, requires specific considerations. In the absence of regional guidelines, a group of experts from across the Gulf region and one international expert from Germany convened to discuss and agree upon a position statement for venetoclax-based fixed-duration treatment strategies for Arab patients with CLL. Our position is that ibrutinib-venetoclax should be the first choice as first-line therapy for all fit CLL patients in the region, regardless of age. The advantages of an all-oral, fixed-duration treatment are discussed in the context of a young Arab patient population, including excellent patient and physician convenience, limited accumulative risk of toxicities, uncomplicated logistics, and low burden of healthcare administration costs. Finally, we discuss the management of key safety considerations in Arab populations including ethnic neutropenia, risk of cardiotoxic events, considerations during intermittent fasting, and avoiding adverse drug–drug interactions, e.g., with anti-tuberculosis or anti-obesity medications.

## Introduction

Chronic lymphocytic leukemia (CLL) is characterized by a slow but progressive accumulation of clonal, mature, dysfunctional B cells that primarily affect the blood, bone marrow and lymph nodes (1). CLL is the most common leukemia in Western countries, comprising 25–30% of all leukemias, with a considerable burden including an incidence of >100,000 cases and > 40,000 deaths per year globally (2). However, its regional prevalence is highly variable, with a substantially lower incidence observed throughout Asia and Africa, comprising as little as 3% of all leukemia cases in the Middle East (2, 3). As such, data on CLL are sparse in Arab populations (4), and as most clinical trials have enrolled Western patients, differences in demographic and genetic backgrounds indicate that established standard clinical parameters need to be adjusted when considering treatment in Arab populations. Patients from the Middle East region tend to be younger, with a median age at diagnosis of 59 years compared with 70 years in the West, and have different baseline comorbidity and hematological profiles (5–7). Therefore, a different clinical management strategy is required for Arab patients with active disease to that which is used for Western patients. In this article, we discuss the utility of venetoclax-based fixed duration (FD) treatment regimens for previously untreated CLL and review the key clinical data in this regard, with a particular focus on the combination of ibrutinib plus venetoclax and obinutuzumab plus venetoclax, both of which have been approved for use in this setting. We highlight the advantages of a FD treatment strategy in the context of a young Arab patient population, and in the absence of regional treatment guidelines, we discuss what we believe to be the most important considerations when selecting and treating Arab patients with a time-limited treatment strategy.

### CLL treatment landscape

The CLL treatment landscape has been revolutionized in recent years, with a wide range of novel treatments available including Bruton tyrosine kinase inhibitors (BTKis), anti-apoptopic protein B-cell lymphoma 2 (BCL-2) inhibitors, phosphoinositide 3-kinase (PI3K) inhibitors, and anti-CD20 monoclonal antibodies (1, 8). The BTKis are highly effective as once- or twice-daily continuous treatments, such that traditional chemoimmunotherapy regimens including rituximab with either fludarabine and cyclophosphamide (FCR) or bendamustine (BR) are no longer widely used (8). Choice of initial therapy depends on a number of factors, including clinical stage, tumor genetic risk stratification [particularly 17p deletion and/or *TP53* and immunoglobulin heavy-chain variable region (*IGHV*) mutational status], patient age, fitness, and presence of comorbidities (1, 8). In particular, cardiac/renal history and concomitant medications are important, as are patient-specific factors such as financial and logistic considerations, as well as individual treatment preferences (9). Single-agent BTKis such as ibrutinib, and more recently acalabrutinib and zanubrutinib, have proven highly effective as once- or twice-daily continuous treatments until disease progression or occurrence of toxicity, and are widely recommended as first-line treatment for CLL (8). While continuous therapies continue to be used with clinical success in CLL, deep remissions are rarely achieved, and potential drawbacks associated with long-term continuous treatment include development of resistance mutations, financial costs, and patient compliance (10). As such, there has been considerable interest in FD treatments, which are able to circumvent some of the problems associated with continuous therapy, and which offer a time-limited treatment option that can limit the development of toxicities that may otherwise accumulate over time. Given CLL is considered an incurable disease, an effective treatment plan is needed that considers the patient’s baseline age and the potential lifelong nature of the disease, while minimizing overall treatment burden on the patient and the impact on their quality of life (11). In this regard, several trials have demonstrated remarkable success of the FD treatment approach in treatment-naïve CLL, particularly combinations of the BCL-2 inhibitor venetoclax alongside ibrutinib (ibrutinib-venetoclax) (12, 13) or the anti-CD20 inhibitor obinutuzumab (obinutuzumab-venetoclax) (14–16).

### Fixed duration treatment: ibrutinib-venetoclax

The rationale for combining ibrutinib with venetoclax is based on their complementary mechanisms of actions, whereby BTKis shrink nodal disease by releasing malignant cells out of lymph nodes into peripheral blood, subsequently allowing venetoclax to actively induce apoptosis of these cells and clear them from the peripheral blood and bone marrow (8, 13, 17, 18). Additionally, ibrutinib sensitizes CLL cells to venetoclax-mediated BCL-2 inhibition, thus allowing a further synergy of the treatment combination (19).

CAPTIVATE, a phase 2 trial in patients aged ≤70 years with previously untreated CLL evaluated treatment with ibrutinib-venetoclax (13). The FD cohort received 3 cycles of ibrutinib lead-in (420 mg once daily), followed by venetoclax (400 mg once daily) for 12 cycles, after a standard 5-week ramp-up. Overall, 92% of patients completed the oral FD combination treatment, with a complete response (CR) rate of 56% in patients without del(17p) and 55% in the all-treated population (13). Undetectable minimal residual disease (MRD) rate was achieved by 77%, with 24-month progression-free (PFS) and overall survival (OS) rates of 95 and 98%, respectively (13). A post-hoc exploratory analysis suggested the deep responses were maintained and comparable outcomes were seen in patients with or without high-risk genetic features, including del(17p), *TP53* mutated, or unmutated *IGHV* (20).

The most common grade ≥ 3 adverse event (AE) in CAPTIVATE was neutropenia (33%), followed by hypertension (6%), with one fatal AE of sudden death during the ibrutinib lead-in (13). Cardiovascular events associated with BTKis occurred at a reduced incidence, likely due to shorter treatment duration (13, 21). Any-grade atrial fibrillation occurred in 4%, and any-grade bleeding events were observed in 61% of patients, with grade 3 and 4 events in 0 and 2% of patients, respectively (13). Tumor lysis syndrome (TLS) risk with venetoclax was mitigated by ibrutinib’s 3-cycle lead-in, reducing high-risk TLS classification in 94% of patients and eliminating TLS events per Howard criteria (13, 22). Only 16% of patients required TLS-hospitalization, easing monitoring and prophylaxis burdens (13, 22).

Another finding of note from CAPTIVATE was the absence of typical resistance mutations to BTKi or BCL2-i after FD treatment with ibrutinib-venetoclax. After 38 months of follow up, 15% of patients who completed FD treatment developed progressive disease (PD), with no significant association with baseline genetic risk factors or resistance mutations in *BTK*, *PLCG2*, or *BCL2* at progression (23). Most patients with PD (19 of 29 patients) were successfully re-treated with either single-agent ibrutinib (*n* = 16) or ibrutinib-venetoclax (*n* = 3), supporting the hypothesis that combination regimens may reduce resistance risk and allow effective re-treatment after PD (23).

While the CAPTIVATE trial enrolled a relatively young, fit CLL patient population, the Phase 3 GLOW study investigated ibrutinib-venetoclax in older and/or less fit comorbid patients (≥65 years or 18–64 years with Cumulative Illness Rating Scale (CIRS) ≥6) (12). Patients received either the same 12-cycle ibrutinib-venetoclax FD regimen as in CAPTIVATE (following the 3-cycle ibrutinib lead-in), or six cycles of chlorambucil-obinutuzumab. The FD ibrutinib-venetoclax combination showed significantly improved PFS versus chemoimmunotherapy (hazard ratio 0.216) (12). Greater undetectable MRD was achieved with ibrutinib-venetoclax at 3 months in both bone marrow (51.9 vs. 17.1% with chlorambucil-obinutuzumab) and peripheral blood (54.7 vs. 39.0%) (12). At a 4-year follow-up, PFS (74.6 vs. 24.8%) and OS (87.5 vs. 77.6%) rates favored ibrutinib-venetoclax, suggesting long-term benefit of this combination after the end of the FD treatment period (24).

In the primary safety analysis in GLOW, the ibrutinib-venetoclax combination resulted in slightly more patients with grade ≥3 AEs compared to chlorambucil and obinutuzumab (75.5 vs. 69.5%), with neutropenia being the most common (34.9 vs. 49.5%). The AE profile may have been influenced by the greater number of patients with multiple comorbidities in the ibrutinib plus venetoclax group (12). All-cause mortality was similar between treatment arms, but four on-treatment cardiac/sudden deaths occurred in the ibrutinib-venetoclax group, all patients with a CIRS score >10 and an Eastern Cooperative Oncology Group Performance Status (ECOG PS) of at least 2 (12). These findings alongside the general risk of cardiac events with BTKi warrant investigation into improved predictive biomarkers for cardiac events among elderly and/or comorbid CLL patients receiving BTKi treatment (12). Of note however, at 4-year follow-up, the number of overall deaths were twice higher in patients receiving chemoimmunotherapy than those receiving ibrutinib-venetoclax, resulting in a significant OS advantage of ibrutinib-venetoclax vs. chemoimmunotherapy (HR 0.48) (24).

The results of CAPTIVATE and GLOW expand upon earlier phase 2 studies that investigated two-year time-limited treatment strategies with ibrutinib-venetoclax in previously untreated, older and/or high-risk patients (25, 26), and demonstrated clinically relevant efficacy of the combination with manageable safety, with an all-oral, 15-month FD treatment that had a favorable benefit–risk ratio which was particularly apparent in the fit patient population.

### Fixed duration treatment: obinutuzumab-venetoclax

The CLL14 trial compared obinutuzumab-venetoclax to chlorambucil-obinutuzumab as first-line treatment of patients with CLL and existing comorbidities (i.e., CIRS of >6) (14). At 24 weeks, obinutuzumab-venetoclax showed superior PFS (88.2 vs. 64.1%), higher undetectable MRD rates in both peripheral blood (75.5 vs. 35.2%) and bone marrow (56.9 vs. 17.1%), and more patients achieved a CR (49.5 vs. 23.1%) compared to chemoimmunotherapy (14). This trend was observed in patients with/without risk factors such as *TP53* mutations or unmutated *IGHV*, but PFS was considerably shorter in these patients regardless of venetoclax or chlorambucil treatment (14). At long-term follow-up (median 39.6 months), median PFS was not reached for obinutuzumab-venetoclax versus 35.6 months for chemoimmunotherapy (27).

The most common grade ≥3 AEs in the obinutuzumab-venetoclax arm were neutropenia (52.8%), thrombocytopenia (13.7%), and infusion-related reactions (9.0%). TLS was observed in three patients in the venetoclax-obinutuzumab group and five patients in the chemoimmunotherapy group, with none of the events meeting Howard criteria. After treatment completion, there were 11 fatal AEs in the obinutuzumab-venetoclax group compared with 4 in the chemoimmunotherapy group (14).

More recently, the CLL13 study compared obinutuzumab-venetoclax ± ibrutinib with rituximab-venetoclax or chemoimmunotherapy in previously untreated fit patients with CLL (median age 60–62 years, with a median CIRS of 2) (16). At 15 months, undetectable MRD in peripheral blood was 52.0% (chemoimmunotherapy) vs. 57.0% (rituximab-venetoclax) vs. 86.5% (obinutuzumab-venetoclax) vs. 92.2% (obinutuzumab-venetoclax-ibrutinib), with CR rates at 15 months of 31.0, 49.4, 56.8, and 61.9%, respectively (16). PFS was significantly improved with obinutuzumab-venetoclax ± ibrutinib except in *IGHV* unmutated patients, who achieved very high PFS in all treatment groups. Early treatment discontinuation due to AEs was highest in the chemoimmunotherapy group (15.3%), followed by the triple therapy obinutuzumab-venetoclax-ibrutinib group (12.6%) (16).

### Considerations for treatment strategies

With multiple highly effective therapeutic options and treatment strategies now available, the choice of appropriate first-line therapy should be based on several disease-related and patient-related factors. The 2025 NCCN guidelines advocate targeted therapies as the primary treatment for CLL patients with 17p deletion or TP53 mutations, based on evidence of its impact on PFS. The recommended options include BTKis and the BCL2 inhibitor venetoclax combined with obinutuzumab (28). The ELEVATE-TN trial demonstrated that acalabrutinib ± obinutuzumab, resulted in notable PFS improvements in patients with del(17p) or TP53 mutations. The estimated 72-month PFS rate was 56% for both treatment arms. Projected 72-month OS rates were 68% for acalabrutinib alone, 72% for acalabrutinib plus obinutuzumab, and 53% for chemoimmunotherapy (29). The SEQUOIA study, a non-randomized trial, prospectively enrolled 111 CLL patients with del(17p) or TP53 mutations. In this group, zanubrutinib monotherapy outperformed bendamustine plus rituximab, showing higher overall response rate (ORR) and substantial PFS improvements. For patients with high del(17p) levels (≥20%), zanubrutinib achieved a 98% ORR and an 89% 18-month PFS rate. In those with low del(17p) (7–20%), the ORR was 92%, with an 88.9% 18-month PFS (30). In lower-risk patients, venetoclax-based FD regimens are likely to be a good option. Considerations between FD regimens may include risk of TLS and barriers to infusion-based therapy (where ibrutinib-venetoclax may be preferred), or uncontrolled cardiac comorbidities and receiving anticoagulation plus antiplatelet therapy (where obinutuzumab-venetoclax may be preferred). The ongoing phase 3 CLL17 trial (NCT04608318) is investigating non-inferiority of FD ibrutinib-venetoclax or obinutuzumab-venetoclax compared with standard-of-care single-agent continuous BTKi inhibitor in first-line treatment of CLL and is anticipated to provide direct head-to-head comparisons on the relative efficacy and safety of FD treatments compared with continuous BTK inhibitor monotherapy.

## Methodology

A group of experts from across the Gulf region of the Middle East and one international expert from Germany convened twice in June 2024, once virtually and once in person in Doha, Qatar. The expert group comprised eight hematologists and one pharmacist who were selected due to their recognized seniority and expertise in the management of CLL. During these meetings the expert group collectively discussed and agreed upon a position statement for venetoclax-based fixed-duration treatment strategies and proposed specific considerations for treatment of Arab patients with CLL. The experts individually took responsibility for the development of specific sections of this article, which were compiled and circulated among all experts for critical review and revisions in a timely manner following the expert meetings.

### First-line, fixed-duration, venetoclax-based treatment strategy for CLL patients in the Middle East

To aid treatment decisions for CLL in the Middle East region, we have developed a position statement based on our personal clinical experience. Our preference for initial treatment with ibrutinib-venetoclax is based on its suitability in patients with a low-risk profile who favor a FD therapy, are averse to infusion therapy, and/or where infusion therapy is deemed too risky from the perspective of the treating physician. This regimen is also our first choice for high-risk patients (e.g., patients with 17p deletion or *TP53* mutations) who are not suitable for continuous BTK inhibitor treatment or are in favor of FD oral therapy. As ibrutinib-venetoclax is an oral therapy, patients should have a good understanding of their therapeutic regimen and common AEs.

### Position statement

Ibrutinib-venetoclax should be the first choice as first-line therapy for all fit CLL patients regardless of age. Ibrutinib-venetoclax should be selected regardless of any cytogenetic high-risk features. Caution should be exercised when considering patients with cardiac comorbidities for treatment with ibrutinib and referral to cardio-oncology or cardiology is advised for risk assessment. Patients with uncontrolled cardiac disease are not candidates for treatment with ibrutinib.

Obinutuzumab-venetoclax should be selected for patients who cannot use ibrutinib-venetoclax. For these patients, obinutuzumab-venetoclax should only be prescribed if the patient can tolerate the therapy. Obinutuzumab-venetoclax is not a first-choice treatment in patients with high-risk features (including unmutated IGHV, del(17p) or TP53 mutation). Obinutuzumab-venetoclax should not be used in patients with active hepatitis B infection.

### Safety considerations in Arab populations

There are several important safety considerations when selecting a FD treatment strategy in Arab patients in the Middle East region. These may differ from treatment strategies in other regions due to unique clinical, socio-economic, and cultural factors in the region, and we have summarized some of the key considerations below.

#### Ethnic neutropenia

One of the most common AEs in patients receiving venetoclax-based FD treatment regimens is neutropenia, which in clinical trials was grade ≥3 in around a third of patients treated with ibrutinib-venetoclax (12, 13) and in around half of patients treated with obinutuzumab-venetoclax (14, 16). Guidance for the management of neutropenia in the prescribing information is based largely on clinical trial data from Western populations; however, there are key differences in hematological profiles between Arab and Western populations that need to be considered when classifying neutropenia and baseline blood cell counts (6, 7). The prevalence of baseline neutropenia is around 11% in Middle Eastern populations, with a higher prevalence seen in ethnic Arab females versus males (32 vs. 6%) (7). As such, we propose three distinct patient categories to guide neutropenia management in Arab CLL populations. Category A includes those patients without neutropenia at baseline, who should be managed according to the standard label recommendations related to dose adjustments. Category B includes patients who have a complete blood cell (CBC) count prior to CLL diagnosis that is consistent with ethnic neutropenia, and who should be managed according to their baseline level of neutropenia regarding assumptions for recovery after dose adjustment. Lastly, Category C encompasses patients for whom no prior CBC data are available prior to the diagnosis of CLL. In these cases, it is essential to establish a family history of neutropenia and medical professionals should exercise clinical judgment when determining whether dose adjustments or interruptions are necessary to manage neutropenia. The hematological reference ranges for white blood cells, absolute neutrophil count, hemoglobin, and platelet count for ethnic Arab patients are shown in Table 1 (6) and should serve as a useful guide for expected baseline ranges in healthy populations.

**Table 1:** Hematological reference ranges for healthy Arabs.

		Age (years)	Hb (g/dL)	WBC	Platelets	ANC
Arab females (*n* = 225)	Mean	39.02	12.740	5.950	260.920	2.780
	SD	12.13	0.970	2.100	59.490	1.760
Arab males (*n* = 515)	Mean	34.76	14.740	6.510	250.670	3.170
	SD	11.48	1.270	2.110	65.210	1.060
Asian females (*n* = 130)	Mean	39.57	12.830	5.990	264.960	2.840
	SD	12.7	0.960	2.100	61.800	1.690
African females (*n* = 95)	Mean	36.15	12.350	5.740	243.120	2.520
	SD	8.69	0.940	2.000	43.780	2.010
Asian males (*n* = 355)	Mean	36.59	14.880	6.720	252.380	3.740
	SD	14.43	1.260	2.020	65.650	1.240
African males (*n* = 160)	Mean	34.19	14.510	6.140	247.700	3.170
	SD	9.75	1.240	2.210	64.340	2.060
Asian young males (*n* = 222)	Mean	28.74	14.990	6.860	259.990	3.720
	SD	5.2	1.190	2.080	65.540	1.700
Asian old males (*n* = 133)	Mean	55.15	14.540	6.320	229.650	3.790
	SD	3.47	1.390	1.800	60.520	2.370
African young males (*n* = 99)	Mean	30.79	14.550	6.240	252.890	3.240
	SD	3.88	1.250	2.200	62.280	2.140
African old males (*n* = 61)	Mean	56.53	14.250	5.450	209.570	2.610
	SD	6.79	1.140	2.120	66.360	1.180
Asian young females (*n* = 87)	Mean	28.76	12.810	6.980	276.760	3.180
	SD	6.86	1.060	6.500	67.300	1.790
Asian old females (*n* = 43)	Mean	50.68	12.840	4.920	252.840	2.490
	SD	5.94	0.850	1.950	52.920	1.490

ANC, absolute neutrophil count; CI, confidence interval; Hb, hemoglobin; SD, standard deviation; WBC, white blood cell. Based on data previously published in Yassin et al. (6).

#### Risk of TLS

TLS is a life-threatening risk that can be associated with venetoclax therapy due to reactivation of apoptosis and rapid lysis of CLL lymphocytes, resulting in the release of tumor cell contents that can lead to multiorgan failure (31, 32). Early clinical trials identified TLS as a venetoclax-associated AE that in some cases could be associated with fatality (33), meaning prevention strategies had to be quickly adopted including the introduction of a gradual dose ramp-up strategy over 5 weeks, prophylactic and monitoring protocols, and patient TLS risk stratification (34). Tumor debulking strategies with an ibrutinib lead-in before venetoclax initiation have also proven effective at mitigating the risk of TLS when treating with ibrutinib-venetoclax in both fit and high-risk patients (22). Recent evidence has emerged to suggest that a 5-week ramp-up of venetoclax may not be a necessary strategy (35); however, while we await further studies to strengthen these data, we continue to recommend the ramp-up protocols. While the general management strategies can be used regardless of ethnicity, special considerations are needed for patients undergoing intermittent fasting, for example during the holy month of Ramadan, because aggressive hydration is needed to limit the risks of life-threatening consequences of TLS, and fasting can interfere with the hydration goal (36). In addition, low urinary output as a result of intermittent fasting can limit the kidney’s ability to counteract the metabolic imbalances during tumor lysis, potentially increasing the risk of negative consequences of TLS (36). Therefore, an additional risk assessment is needed in patients who are committed to practicing intermittent fasting and are due to be initiated on venetoclax.

#### Peptic ulcer disease and risk of gastrointestinal bleeding

BTK inhibitors are also associated with an increased risk of bleeding events due to their potential for on- and off-target inhibition of Tec family kinases in thrombocytes that mediate platelet signaling (37). A small but sizeable proportion (4%) of patients in clinical trials receiving ibrutinib experienced major hemorrhage, including gastrointestinal (GI) bleeding, especially when there is comedication with anticoagulants (36). This rate may be slightly higher with ibrutinib than for other BTK inhibitors, possibly due to the wider spectrum of off-target activity seen with ibrutinib (38). GI bleeding can be caused or exacerbated by peptic ulcer disease (PUD), which is an additional concern in patients from the Midde East region who have a relatively high prevalence of PUD (39). Increases in bleeding and PUD complications due to increased gastric acid levels can also occur during the holy month of Ramadan (36, 40, 41), suggesting that fasting may be associated with increased risk of hemorrhage and should therefore be taken into consideration when designing an appropriate treatment strategy. The rate of PUD in the Middle East also means that patients are often receiving proton-pump inhibitors (PPIs) or histamine H2 receptor antagonists (H2RAs). Drug interactions can lead to lack of efficacy of one or both agents via changes in active plasma concentrations due to alterations in drug absorption, distribution, metabolism, and excretion, or indeed can lead to new or exacerbated adverse reactions, by direct effects on drug metabolism. Additionally, effects can be via direct interference with biological or physiological drug function (42). We have compiled potential drug–drug interactions of PPIs/H2RAs with common CLL treatments, along with the mechanism and recommended action, and summarized these in Table 2.

**Table 2:** Summary of potential drug–drug interactions of CLL treatments with PPIs, H2RAs, and anti-obesity.

Medication(s)	Ibrutinib	Acalabrutinib	Zanubrutinib	Venetoclax
Omeprazole, Lansoprazole, Esomeprazole, Pantoprazole, Rabeprazole	Proton Pump Inhibitors			
	Effects			
	No effect.	AUC was decreased by 43% when acalabrutinib capsules were co-administered with the PPI.	With omeprazole: CYP2C19 inducers (weak) may decrease the serum concentration of omeprazole.	No effect.
	Mechanism			
	–	The mechanism is likely to the reduced solubility of acalabrutinib capsules due to increased gastric pH caused by inhibitors of the proton pump.	The mechanism is likely due to induction of CYP2C19, an enzyme responsible for omeprazole metabolism.	–
	Action			
	–	Consider using the tablet formulation.	No action required	–
Cimetidine, Famotidine, Lafutidine, Nizatidine, Ranitidine, Famotidine	Histamine H2 Receptors Antagonists			
	Effects			
	No effect.	-Formulation dependent: This interaction is only applicable to acalabrutinib capsules. -H2RAs may decrease the serum concentration of Acalabrutinib.	No effect.	No effect.
	Mechanism			
		The mechanism of this interaction is likely reduced solubility of acalabrutinib capsules due to increased gastric pH caused by the gastric acid suppressant.		
	Action			
		Separate administration of H2RAs and acalabrutinib capsules by giving acalabrutinib capsules 2 h before a H2RA		
Liraglutide Semaglutide Tirzepatide	Weight Loss Medications			
	Glucagon-Like Peptide-1 Receptor Agonists			
	Effects			
	No identified drug interactions.			Hyperglycemia-associated agents may diminish the therapeutic effect of antidiabetic agents.
	Mechanism			
	–	–	–	Not identified.
	Action			
	–	–	–	Monitor therapy.
Orlistat	Lipase Inhibitors			
	No identified drug interactions.			
Naltrexone and Bupropion	Opiate Antagonists/Antidepressants			
	No identified drug interactions.			
Phentermine and Topiramate	Sympathomimetic Amine Anorectic/Antiepileptic Analog			
	No identified drug interactions.			

AUC: area under the curve; C_max_, maximum concentration; CLL, chronic lymphocytic leukemia; CYP, cytochrome; H2RA, Histamine H2 Receptors Antagonists; PPI, proton pump inhibitor. Drug interactions analysis was performed using Lexicomp, an online database that combines medical literature and scientific understanding of drug interactions in an electronic platform, providing an efficient way to inform professionals about adverse drug events that could compromise the care of patients.

#### Tuberculosis and concomitant use of anti-tuberculosis agents

CLL increases patient susceptibility to infections, with factors such as immune dysregulation, *IGHV* status, and treatment, contributing to higher infection rates (43). Tuberculosis (TB) is also endemic in the Middle East and Arab nations, with a reported incidence of 26.79 per 100,000 across the Middle East and North Africa in 2019 (44). Commonly used anti-tuberculosis medications can alter pharmacokinetic parameters of co-medications by direct effects on liver metabolism (45), therefore devising a treatment strategy for CLL patients taking anti-TB medications has to take this into consideration. To help physicians in the region circumvent such drug–drug interactions, we have checked for potential interactions between first- and second-line anti-TB medications and summarized these, along with our recommendations, in Table 2.

#### Risk of adverse cardiotoxic events

Cardiac AEs are a class effect that can occur with all available BTKis (46–48), and are thought to be mediated by co-inhibition of BTK and homologous non-receptor tyrosine kinases within cardiomyocytes (48). Physicians should exercise caution in concluding that second-generation BTKis are safer than ibrutinib in this regard (46). While the majority of such cardiac AEs are low grade, high-grade, life-threatening events do occur (47). Trials with stringent cardiac observations of patients have resulted in low rates of such BTKi-related toxicity (49). This emphasizes the need to address this issue in all patients undergoing such treatment, particularly in patients with cardiac comorbidities who have a 3–4-fold higher risk of developing cardiac AEs upon BTKi treatment (50). This is particularly relevant for Middle Eastern populations, who have a substantial burden of cardiac comorbidities, including high levels of diabetes (51), obesity (52), hyperlipidemia (53), and uncontrolled hypertension (54). It is therefore essential to identify patients with such baseline risk, optimize cardiac disorder-related medication before BTKi initiation, and monitor at-risk patients accordingly while on treatment. This includes regular clinical examination and ECG evaluation every 3 months or upon any signs and symptoms suggestive of heart-related origin, and patients should be advised accordingly. Furthermore, we recommend regular cardiology visits to monitor current cardiac status and related medication. Individuals with poorly controlled heart disease should not receive BTKis as first-line CLL therapy, neither continuous nor FD, and patients on BTKis who experience cardiovascular or other bothersome AEs should undergo dose modification. This approach frequently resolves and reduces reoccurrence of the event and enables patients to remain on drug for a longer period of time, and several prospective and retrospective studies suggest that such dose reductions for AE management do not impair the efficacy of treatment (55–59). It is important to consider potential drug interactions of CLL treatments with anti-obesity agents, due to the high prevalence of obese and overweight individuals (21.17 and 33.14, respectively) across the Middle East region (60). We have summarized potential drug–drug interactions of CLL treatments with anti-obesity agents, along with mechanisms and recommendations to avoid interactions in Table 3.

**Table 3:** Summary of potential drug–drug interactions of CLL treatments with anti-tuberculosis agents.

Medication(s)	Ibrutinib	Acalabrutinib	Zanubrutinib	Venetoclax
Rifampicin	First-line anti-tuberculosis agent			
	Effects			
	Decrease the ibrutinib C_max_ and AUC by 92% and 90%, respectively.	Decrease the acalabrutinib C_max_ and AUC by 68% and 77%, respectively.	Decrease the zanubrutinib AUC and C_max_ by 93% and 92%, respectively.	Decrease the venetoclax C_max_ and AUC by 42% and 71%, respectively.
	Mechanism			
	Strong induction of CYP 3A4 by rifampin.			
	Action			
	Concomitant use of ibrutinib and strong CYP3A4 inducers is not recommended.	-Concomitant use of acalabrutinib and strong CYP3A4 inducers should be avoided when possible. -If combined, the acalabrutinib dose should be increased to 200 mg twice daily.	Avoid coadministration of zanubrutinib with strong CYP3A4 inducers.	Avoid concomitant use of strong CYP3A4 inducers with venetoclax.
Isoniazid	Effects			
	No effect found.	No effect found.	CYP3A4 Inhibitors (Weak) may increase the serum concentration of zanubrutinib.	No effect found.
	Mechanism			
	–	–	Inhibition of CYP3A4, an enzyme responsible for zanubrutinib metabolism.	–
	Action			
	–	–	No action required beyond standard clinical care measure.	–
Rifabutin	Effects			
	Serum concentration may decrease. The interaction with strong CYP3A4 inducer rifampin decreased the ibrutinib C_max_ and AUC 92% and 90%, respectively.	No studies have evaluated the effects of moderate CYP3A4 inducers on the PK of acalabrutinib. However, the C_max_ and AUC were reduced by 68% and 77%, respectively, in healthy subjects when given concurrently with the strong CYP3A inducer rifampin.	Decreased the zanubrutinib AUC and C_max_ 44% and 48%, respectively.	Clinical data characterizing the impact of moderate CYP3A4 inhibitors on venetoclax exposure are not available. However, with strong CYP3A inducer. C_max_ and AUC of venetoclax were reduced by 42% and 71%, respectively.
	Mechanism			
	Induction of CYP 3A4.			
	Action			
	Monitor for decreased ibrutinib effects/therapeutic failure if combined with moderate CYP3A4 inducers.	Monitor for reduced acalabrutinib efficacy when combined with moderate CYP3A4 inducers.	Avoid coadministration of zanubrutinib with moderate CYP3A4 inducers if possible. If coadministration is required, increase the zanubrutinib dose to 320 mg twice daily and resume the previous zanubrutinib dose after the moderate CYP3A4 inducer has been discontinued.	Prescribing information recommends avoiding use with strong or moderate CYP3A4 inducers.
Pyrazinamide	No identified drug interactions.			
Streptomycin				
Ethambutol				
Amikacin	Second-line anti-tuberculosis agent			
	No identified drug interactions			
Amoxicillin /Clavulanate				
Bedaquiline				
Clofazimine	Effects			
	Clofazimine may increase the serum concentration of CYP3A4 substrates.	Clofazimine may increase the serum concentration of CYP3A4 substrates.		
	Mechanism			
	No clinical studies have evaluated the effects of clofazimine on the PK of CYP3A4 substrates. However, *in vitro* data suggests that clofazimine may be a moderate to strong CYP3A4 inhibitors			
	Action			
	Monitor for increased toxicities/effects of CYP3A4 substrates if combined with clofazimine	Monitor for increased toxicities/effects of CYP3A4 substrates if combined with clofazimine.		
Delamanid	No identified drug interactions			
Cycloserine				
Ethionamide				
Imipenem and Cilastatin				
Kanamycin				
Levofloxacin				
Meropenem				
Linezolid	Effects			
	Linezolid may enhance the myelosuppressive effect of myelosuppressive agent.			
	Mechanism			
	The mechanism of this interaction is additive myelosuppressive effects.			
	Action			
	Monitor complete blood counts weekly in patients receiving linezolid with other agents capable of causing significant myelosuppression.			
Pretomanid	Effects			
	No effect found.		Pretomanid may increase the serum concentration of P-glycoprotein Substrates (narrow therapeutic index/sensitive with inhibitors).	
	Mechanism			
	–		The mechanism for any such interaction would be inhibition of P-gp-mediated transport of P-gp substrates.	
	Action			
	–		Monitor closely for evidence of altered response to a P-glycoprotein substrate during treatment with pretomanid.	
Moxifloxacin	No identified drug interactions.			
Rifapentine	Effects			
	CYP3A4 inducers (moderate) may decrease the serum concentration of ibrutinib, acalabrutinib, zanubrutinib and venetoclax.			
	Mechanism			
	Due to induction of CYP3A4		The suspected primary mechanism of this interaction is rifampin induction of CYP3A4-mediated venetoclax metabolism. Clinical data characterizing the impact of moderate CYP3A4 inhibitors on venetoclax exposure are not available. Venetoclax prescribing information recommends avoiding use with strong or moderate CYP3A4 inducers.	
	Action			
	No recommendation is provided for when ibrutinib is combined with moderate CYP3A4 inducers, but reduced exposure to ibrutinib appears likely and monitoring for reduced effectiveness is warranted.	No recommendations are provided for use with moderate CYP3A4 inducers, but increased monitoring for reduced acalabrutinib efficacy is warranted when these agents are combined.	If coadministration with moderate CYP3A4 inducers cannot be avoided, increase the zanubrutinib dose to 320 mg twice daily during coadministration, and resume the previous zanubrutinib dose after discontinuation of the moderate CYP3A4 inducer.	Avoid concomitant use of moderate CYP3A4 inducers with venetoclax.

AUC: area under the curve; C_max_, maximum concentration; CLL, chronic lymphocytic leukemia; CYP, cytochrome; P-gp, P-glycoprotein; PK, pharmacokinetics. Drug interactions analysis was performed using Lexicomp, an online database that combines medical literature and scientific understanding of drug interactions in an electronic platform, providing an efficient way to inform professionals about adverse drug events that could compromise the care of patients.

#### Renal impairment

Renal insufficiency is an important factor in treatment decisions and clinical management. The incidence of chronic kidney disease (CKD), the risk of which is increased in the presence of other comorbidities including diabetes, obesity and hypertension, is increasingly common in the Middle East (61). The presence of CKD requires certain dose adjustments, with consideration of tumor lysis risk, that varies by disease stage, and should be closely monitored for the potential impact on drug–drug interactions.

#### Fertility and pregnancy

Pregnancy and fertility-related concerns are important in the treatment planning and monitoring of patients with CLL in the Middle East region, given the younger age of patients at diagnosis, compared to Western patients (5). Unlike the well-documented effects of traditional chemo-immunotherapies, very little is known about the impact of BTKis and BCL2 antagonists on reproductive health, and there are no data on the effect of these agents on fertility or to guide the use of these agents during pregnancy. Female patients of reproductive age as well as male patients should all be counseled on the potential risk of infertility and advised about methods of fertility preservation prior to the initiation of therapy (32, 62, 63). All female patients with reproductive potential should be tested for pregnancy prior to initiation of therapy, and given the potential for fetal harm, the use of effective contraception is recommended in these patients while receiving therapy. Similarly, in the absence of data on the presence of these agents or their metabolites in breast milk, lactating women are advised not to breastfeed during treatment to avoid potential AEs in the breastfed child. Female patients are advised to avoid becoming pregnant for at least 30 days from last dose of these agents, and male patients are advised not to father a child while receiving treatment or for at least 10–30 days from last dose (62, 63).

## Discussion

**Figure 1:**
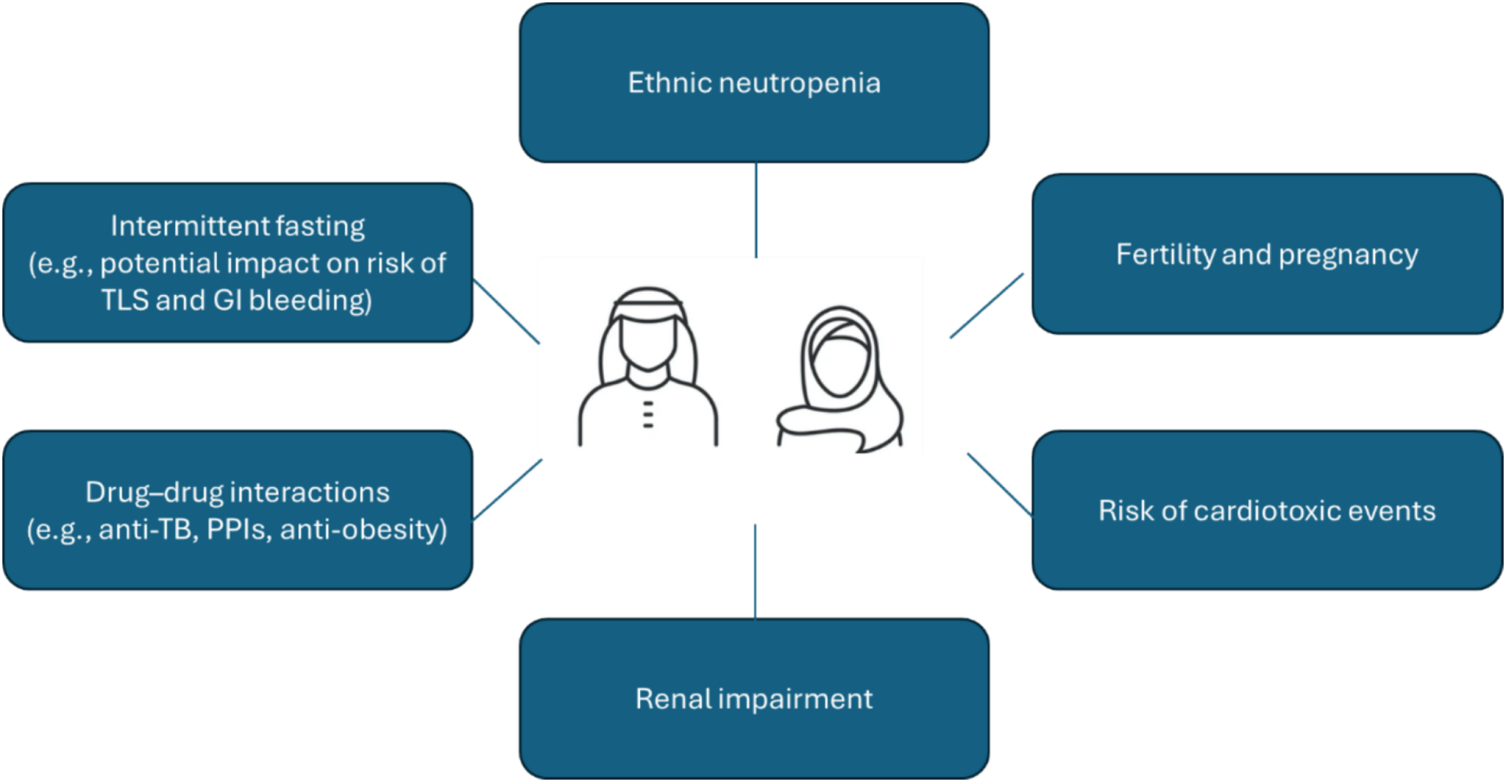
Key safety considerations for CLL patients in the Middle East region. CLL, chronic lymphocytic leukemia; GI, gastrointestinal; PPI, proton pump inhibitors; TB, tuberculosis; TLS, tumor lysis syndrome.

FD treatment strategies have the potential to offer several advantages over continuous therapy for patients from Arab nations. Firstly, Arab patients tend to be approximately 10 years younger than Western patients at diagnosis (5), meaning effective alternatives to potentially lifelong continuous therapies are even more important from both patient and clinical perspectives. Time-limited treatments in a younger population can effectively limit the cumulative risk of toxicity associated with continuous treatment, particularly with respect to potential cardiac toxicities that are known to be associated with long-term BTKi use. This is particularly advantageous in Middle Eastern populations that are susceptible to cardiovascular diseases due to relatively high baseline levels of obesity, diabetes, hypercholesterolemia, and hypertension (51–54). Our position statement for Arab patients is that ibrutinib-venetoclax should be the first choice as first-line therapy for all fit CLL patients regardless of age. It is of note that combination therapies like ibrutinib-venetoclax can produce overlapping toxicities such as TLS, cardiac issues, and cytopenia which necessitate close patient monitoring, and tailored prophylaxis. Figure 1 summarizes what we believe to be the key safety considerations in this population based on our clinical experience.

An additional benefit of FD treatment is the potential for a reduction in treatment costs, where lifetime savings versus continuous treatment would be far greater in Arab patients given they are typically diagnosed with CLL approximately 10 years younger than their Western counterparts (5). This necessitates a particular emphasis on limiting the cumulative risk of toxicity associated with lifelong therapies, especially the potential cardiac toxicity linked to BTKis (64). This consideration is particularly relevant for Middle Eastern populations, who exhibit a higher susceptibility to cardiovascular diseases due to the relatively high prevalence of baseline obesity, diabetes, and hypertension (65).

From a health economics perspective, fixed-duration ibrutinib-venetoclax as an all-oral regimen should be the treatment of choice for indicated CLL patients in the front-line setting. Ongoing work demonstrates the all-oral regimen leads to cost savings including drug costs, direct medical costs, direct non-medical costs, and indirect costs. This comprises lower utilization of healthcare resources including daycare unit visits, nursing time, pharmacy time, intravenous fluids, medical consumables, transportation time, and home carers, in addition to reductions in absenteeism and presenteeism (66). Furthermore, the majority of CLL patients in the Gulf region are expatriates whose families depend on their incomes (66). This underscores the necessity for well-tolerated therapies that not only improve overall quality of life but also minimize work absences, thereby supporting both the patient and their economic stability.

A further benefit of oral therapy over infusion therapy is the lack of infusion-related reactions. These reactions can range from mild to severe and often require close monitoring and supportive care during and after administration, which can be logistically challenging in healthcare settings with limited infusion resources or where access to emergency medical intervention might be delayed. Moreover, in some healthcare systems within the Middle East, logistical barriers such as the availability of infusion centers, patient preference for convenience, and challenges in travel or access to specialized care facilities can make infusion therapy less favorable. These practical considerations often influence the risk–benefit assessment and the preference for an oral FD regimen, which offers efficacy comparable to infusion-based regimens without the associated infusion-related risks. Additionally, being an FD treatment, ibrutinib-venetoclax would have lower cumulative drug costs compared to other available continuous treatment oral options. FD therapy could also positively impact quality of life as patients will have the opportunity to be treatment-free after only 15 months.

When using a FD treatment strategy in the Middle East, it is important to note that most centers rely on clinical assessment to evaluate treatment efficacy, while MRD is still largely viewed as an unvalidated, experimental assessment that is not routinely used. This needs to be addressed across the region, given that MRD is the most effective way to assess the depth of remission and determine the likelihood of patient relapse (67). Flow cytometry is widely available throughout the region, meaning most centers have the capability to directly assess MRD, while next-generation sequencing facilities are also available, albeit in a limited number of specialized facilities. Despite these capabilities, MRD assessment has not been widely adopted in the region due to lack of expertise and validation in Arab patient populations, alongside the lack of standardized local protocols to support its widespread use. At present there is a lack of data on long-term outcomes and sustainability of FD treatment strategies; however, the European research initiative in CLL (ERIC) is initiating a multicenter project to further examine the ibrutinib-venetoclax FD protocol and work is being conducted in several centers across the Gulf region to generate real-world data on the use of this treatment strategy. A focus of future CLL clinical management initiatives in Gulf countries should include education and uptake on the use of MRD, which is an accessible prognostic technique that can be used as an effective tool to guide treatment decisions (67).

## Conclusion

In the absence of large clinical datasets in Arab patients, we have drawn on our collective experience to outline key considerations for clinicians in the Middle East region who are managing CLL patients using a FD treatment strategy. The selection of the optimal treatment strategy depends on a number of factors, most importantly patient demographics, baseline comorbidities, and preferences/logistics. Our position statement is that ibrutinib-venetoclax should be the first choice as first-line therapy for all fit CLL patients in the region, irrespective of their age. In addition, the option of an all-oral treatment with ibrutinib-venetoclax presents several advantages in terms of patient convenience, logistics, and reducing the burden on healthcare administration costs compared with monoclonal antibody-based treatments.

## Data Availability

The original contributions presented in the study are included in the article/supplementary material, further inquiries can be directed to the corresponding author.
